# Nutritional and Functional Properties of Novel Italian Spray-Dried Cricket Powder

**DOI:** 10.3390/antiox12010112

**Published:** 2023-01-02

**Authors:** Marco Ruggeri, Eleonora Bianchi, Barbara Vigani, Rita Sánchez-Espejo, Mattia Spano, Carlotta Totaro Fila, Luisa Mannina, César Viseras, Silvia Rossi, Giuseppina Sandri

**Affiliations:** 1 Department of Drug Sciences, University of Pavia, Viale Taramelli 12, 27100 Pavia, Italy; 2 Department of Pharmacy and Pharmaceutical Technology, Faculty of Pharmacy, University of Granada, Campus of Cartuja s/n, 18071 Granada, Spain; 3 Department of Chemistry and Technology of Drugs, Sapienza University of Rome, Piazzale Aldo Moro 5, 00185 Rome, Italy; 4 Alia Insect Farm, Via Olmetto, 20123 Milan, Italy

**Keywords:** *Acheta domesticus*, chitin, surface zeta potential, antioxidant properties, immune response

## Abstract

Insects, especially crickets, have been proposed as a novel source of nutrients in human nutrition since they possess bioactive molecules, including high protein content, lipids, chitin, vitamins and minerals. In this work, the nutritional and functional properties of a novel Italian spray-dried (SD) cricket powder were evaluated. The powder was characterized by physico–chemical properties (morphology, size distribution, solid state, thermal profiles, and surface zeta potential), and antioxidant properties. Moreover, preclinical properties (cytocompatibility and pro-inflammatory immune response) were assessed. The powder was characterized by microparticle structure with bulges and rough surfaces, showing distinctive antioxidant properties. The preclinical results suggested that the SD crickets were biocompatible towards Caco-2 and macrophages without immune response, representing an interesting material for the food industry that could provide health benefits in addition to the basic nutritional value of traditional foods.

## 1. Introduction

The rapid growth of the world population, which is projected to surpass nine billion people by 2050, means an anticipated rise in demand for animal proteins, with growth for such proteins expected to hit 70–80% [1,2]. A great challenge for the future will be the task of finding a sustainable way to feed an increasing number of demanding consumers. This is an extremely urgent issue, since the current livestock sector is a major emitter of greenhouse gas emissions, responsible (together with related industries) for about 14.5% of total global greenhouse gas emissions [3,4]. Moreover, farm animals are a large contributor to the impoverishment of agricultural land, driving further climate change [4,5]. For these reasons, new ways to produce high-quality proteins are of fundamental importance in the near future to tackle both undernutrition and environmental issues.

Mini-livestocks of edible insects have been proposed as an alternative source of animal proteins, and they are currently considered a sustainable solution to environmental pollution and food-related risk [6]. Although eating insects might seem unappealing, approximately 1900 insect species are eaten worldwide, constituting quality food and feed. Interestingly, they are characterized by high feed conversion ratios, showing twice the efficiency of chickens, four times the efficiency of pigs, and twelve times that of cattle. Moreover, insects require less land, water, and feed to provide an equivalent amount of edible mass, resulting in the generation of fewer greenhouse gases [2]. In particular, the house cricket (*Acheta domesticus*) is on the road to approval as a human food in both the U.S. and European markets [7]. In Europe, the marketing of insects as food is ruled by the “Novel Foods” legislation—Regulation (EU) No 2015/2283, that applies to all categories of foods that “were not used for human consumption to a significant degree” within the European Union before 15 May 1997, which is the case for insects. Presently (Q3 2022), four Novel Food authorizations have entered into force for edible insects, and the EFSA (European Food Security Authority) has published a sixth opinion on edible insects, highlighting that food safety and quality are strictly related to the species and production process [8]. Crickets are highly nourishing and rich in high-quality proteins, providing an excellent source of recommended essential amino acids, with the possible exception of methionine/cysteine. They are also a good source of riboflavin, folic acid, vitamin B12, tocopherols, and several minerals [9].

Moreover, insects have proved to be beneficial for human health because of the presence of bioactive molecules acting as anti-inflammatory, cardioprotective, antimicrobial, antimycotic and antioxidant compounds [10]. In particular, chitin, a bioactive polysaccharide from insect exoskeleton, is characterized by remarkable antioxidant and antimicrobial activities. These could offer a wide range of applications in many sectors, helping protect the population from diseases such as cancer, neurodegenerative and inflammatory diseases [11].

However, despite the numerous advantages, consumers, particularly in the Western sphere, retain an unfavourable disposition towards culinary options that are not traditionally considered to be food, since dietary choice usually takes place within a range of familiar foods. For this reason, the consumption of insects could be promoted by producing a variety of products which differ from the whole insects, including more versatile and acceptable commodities, such as cricket powder [12].

The more common manufacturing method involves grinding whole crickets to a paste, followed by drying in an oven, in order to obtain a “cricket cake”, and finally undertaking particle size reduction in an industrial food processor to obtain a flour. The major issue with the final product obtained through this method is that the particle size often exceeds 300 µm, since the cricket exoskeleton made of chitin resists grinding and remains mostly intact. This is not a problem in the products in which these particle sizes are acceptable, such as bars or some baked goods. However, it could greatly reduce sensory acceptance in other products (such as beverages or puddings), since particles over 100 µm could seem unpleasant to the human tongue and oral palate [13]. To overcome this problem, other processes have been set up to produce a finer cricket powder by wet-blending the cricket mass, and then spray-drying it. This process results in a spray-dried (SD) cricket powder different in color, flavour, and textural properties from “cricket cakes”. In fact, spray-drying is a simple, easily-scalable, and versatile technique which allows microparticles with a size distribution of anything from several μm to several hundred μm [14] to be obtained, resulting in a more pleasant product for the human palate. Moreover, the process can be completed in shorter time periods, allowing the taste to be preserved intact.

Given these premises, this research aims to assess the nutritional and functional properties of a novel Italian SD cricket powder, that could represent an interesting advancement in the food industry by providing health benefits in addition to the basic nutritional value of traditional foods. Solid state characterization, including morphology, was assessed to determine a product fingerprint together with biocompatibility properties. These in vitro characterizations are required to understand the convenience of deepening the in vivo characterization for nutritional clinical translation.

## 2. Materials and Methods

### 2.1. Materials

SD crickets were kindly donated by Alia Insect Farm. Crickets between 35 and 42 days old (one or two stages before the complete adult one) were used. The insects were pasteurized, and then minced and milled to obtain a homogeneous blend. This was spray dried at 200 °C (inlet temperature), producing a fine powder with a residual humidity below 5%. Ten batches were prepared and 50 g samplings from each were studied.

### 2.2. Physico-Chemical Characterization

Scanning electron microscopy (Tescan, Mira3XMU, Brno, Czech Republic) was used to evaluate microparticles’ morphology. SD crickets were put onto stubs and sputtered with carbon under vacuum. 

Particle size distribution was determined using dynamic light scattering (Mastersizer 3000E granulometer, Malvern Instruments, Milan, Italy). A suspension of SD crickets was prepared in isopropanol and particle size parameters were obtained.

Using a JASCO 6200 (USA) outfitted with a Ge ATR, Fourier-transform infrared (FT-IR) analysis was carried out. All investigations were carried out between 400 and 4000 cm^−1^ at a resolution of 2 cm^−1^, and the software Spectra Manager v2 was used to handle the data.

Thermogravimetric analysis (TGA) and differential scanning calorimetry (DSC) were carried out in ambient air, at a temperature range of 25 to 950 °C, and a heating rate of 10 °C/min, using a TGA/DSC1 instrument (Mettler-Toledo GMBH, Madrid, Spain). After this, 20 mg SD crickets were placed in aluminium sample pans and subjected to characterizations.

### 2.3. Chitin Quantification

Acetyl group determination was evaluated after the total hydrolysis of SD crickets according to Hahn et al. [15]. First, 0.25 g of powder (w_BM_) was added in 10 mL of 6 M HCl, and heated at 130 °C for 45 min. After this, 10 mL 6 M NaOH was added, and re-heated for 45 min at 130 °C. The samples were then centrifuged at 4000× *g* and supernatants neutralized with 10 mL of 2 M HCl. Following this, 2 mL of each processed sample was collected, and an enzymatic test was performed using a kit (R-Biopharm AG, Pfungstadt, Germany) to determine the amount of acetyl groups. The content of chitin (*w_chitin_*) was calculated using the mass of acetate found in the supernatant (*m_acetate_*) and the biomass used (*w_BM_*) through the following formula:

(1) $$w_{chitin}\;\left[g/kg\right]=\frac{3.65\;\times \;m_{acetate}\;\left[g\right]}{w_{BM}\left[kg\right]}$$

The factor 3.65 considers the molecular weights of N-acetyl glucosamine, glucosamine, and acetic acid, assuming a degree of acetylation (DA) of 90% (0.9) in insects. Three replicates were performed, each derived from a different batch.

### 2.4. Surface Zeta Potential (ζ)

The ζ of SD crickets was assessed by means of SurPASS™ 3 (Anton Paar, Turin, Italy) using the cylindrical cell. First, 50 mg SD crickets were placed between two filter disks and 0.01 mol/l KCl solution was used as streaming solvent. The pH was scanned from 2 to 9 to determine the ζ and the isoelectric point (iep) was also assessed [16]. Three replicates were performed, and each sample derived from a different batch.

### 2.5. Protein Digestability Assay

The protein quality of SD crickets was evaluated by means of an in vitro Protein Digestibility-Corrected Amino Acid Score (PDCAAS) enzyme digestion method (Megazyme, Wicklow, Ireland). First, 19 mL HCl (0.06 N) was mixed with 500 mg of SD crickets and placed in a shaking bath (150 rpm) for 30 min at 37 °C. Afterwards, 1 mL pepsin solution was added, vortexed and incubated at 150 rpm. After 1 h, the pH of each sample was neutralized at 7.4 using 1M Tris buffer pH 7.4. Samples were vortexed and subsequently a trypsin/chymotrypsin solution (200 µL) was added and re-incubated. After 4 h of trypsin/chymotrypsin digestion, each sample was heated at 100 °C for 10 min. Subsequently, the samples were completely cooled and 40% trichloroacetic acid solution was added to the mixture in a volume ratio of 1:4. Finally, the samples were centrifuged for 10 min at 15,000 rpm and diluted (1:10 volume ratio) in acetate buffer (50 mM, pH 5.5). Ninhydrin (2% *w/v* ninhydrin, hydrindantin 6.8 mg/L in 3:1 *v/v* DMSO: lithium acetate buffer 4 M, pH 5.2; Sigma-Aldrich, Milan, Italy) assay was performed and the optical density at 570 nm was read by means of a FLUOstar^®^ microplate reader. A calibration curve was obtained using L-glycine and the method was linear between 0.005 and 0.75 mM (R^2^ > 0.998). Casein, a highly nourishing protein that is widely consumed, was used as reference. The results were processed using a Megazyme Mega-Calc^TM^ calculator. Ten replicates were performed, each derived from a different batch.

### 2.6. Antioxidant Properties

To evaluate the antioxidant properties in time, 10 mg SD crickets were put in 2 mL of PBS at 37 °C [16,17]. At prefixed time points, the released media were withdrawn, and split in equal volumes. One aliquot was used to evaluate the DPPH activity (direct antioxidant properties), specifically by mixing 0.1 mL of the released media with 0.1 mL of DPPH methanol solution (8 μg/mL). The resulting mixture was incubated for 30 min in the dark and the corresponding absorbance was measured at 515 nm (FLUOstar^®^ Omega, BMG LABTECH, Aylesbury, UK). The results were expressed as DPPH radical scavenging activity and gallic acid equivalents (μg GA/10 mg sample).

The second aliquot was mixed with 0.125 mM FeCl_2_ solution (0.05 mL) in a 1:1 volume ratio, and then 0.3125 mM ferrozine (0.1 mL) was added. After 10 min equilibrium time to allow Fe^2+^ chelation, the absorbance was detected at 562 nm against the blank (sample before the reaction) and the results were expressed as ferrous ion chelating activity and EDTA equivalents (μg EDTA/10 mg sample). The calibration curves of GA and EDTA were linear with R^2^ values always higher than 0.994. Four replicates were performed, each derived from a different batch.

### 2.7. Cell Biocompatibility

Biocompatibility was assessed using two methods: direct and indirect test. In the direct test, the samples were prepared as SD cricket suspensions, while in the indirect test, as extracts. In both tests, the SD crickets were carefully weighed and suspended in DMEM medium (Dulbecco’s Modified Eagle’s Medium, PromoCell, WVR, Milan, Italy) to obtain samples ranging from 0.05 to 2% *w/w*. The suspensions were freshly prepared at different concentrations and vortexed just before contact with the cells to facilitate a homogeneous dispersion, while the extracts were obtained by centrifugation starting from the suspensions after 24 h contact at 37 °C.

Complete medium and Triton^®^X100 (Fluka, Milan, Italy) were used as positive and negative controls, respectively. Caco-2/TC-7 Human Colon Adenocarcinoma Cell Line (Merck, Milan, Italy) were plated in 96-well plates (growth area 0.36 cm^2^, Greineger Bio-one, PBI International, Italy) at a density of 25,000 cells/well and cultured for 24 h (37 °C, 5% CO_2_). Then, the medium was removed and 200 μl cricket extract or suspension at different concentrations was added to each well. After one day, the cell biocompatibility was assessed by means of an MTT [3-(4,5-dimethylthiazol-2-yl)-2,5-diphenyltetrazolium bromide] assay. The medium in each well was discarded and 100 μL of MTT solution at 1 mg/mL in DMEM w/o phenol red (PromoCell, WVR, Italy) was placed. Afterwards, the cells were incubated at 37 °C for 3 h, and isopropanol (Carlo Erba, Italy) was used to dissolve formazan salts. The absorbance was detected using FLUOstar^®^ Omega MicroplateReader (BMG LABTECH, Italy) at λ = 570 nm (with reference λ = 690 nm). Five replicates were performed, each derived from a different batch.

### 2.8. Cytocompatibility of Macrophages and Pro-Inflammatory Immune Response

The cytocompatibility of macrophages (using hMoCD14 + -PB-c cell line, human CD14+ monocytes derived from peripheral blood, Carlo Erba, Italy) and pro-inflammatory immune response were assessed as previously described by Ruggeri et al. [17].

SD crickets (10 mg) were incubated at 37 °C in a serum-free DMEM medium (Dulbecco’s Modified Eagle’s Medium, PromoCell, WVR, Italy) for one day to produce extraction media. The cells were cultured with a seeding density of 20 × 10^3^ cells/well in a 24-well and treated with 100 nM for 1 × 10^5^ cells of phorbol 12-myristate-13-acetate (PMA, Sigma-Aldrich, Italy) to allow differentiation. After one day, extraction media were added to the cell substrates and incubated for one and two days. The SD crickets’ cytocompatibility was assessed by means of MTT assay, detecting absorbance at λ = 570 nm (with reference λ = 690 nm).

Using an ELISA kit, proinflammatory cytokine TNFα was used to measure the proinflammatory immune response (Thermo Fisher, Italy). After one and two days of SD cricket contact, supernatants from the cultures were removed, and the TNFα released by macrophages was measured at 450 nm, with 570 nm as the reference wavelength. As a positive control, lipopolysaccharide (LPS, 10 g/mL for 24 h) was used. Eight replicates were performed, each derived from a different batch.

### 2.9. Statistical Analysis

Statistical analyses were performed using an Astatsa statistical calculator. One-way analysis of variance (ANOVA) was followed by Scheffé for post-hoc comparisons. *p* < 0.05 was considered significant. Shapiro–Wilk tests were used to verify if the variables were normally distributed.

## 3. Results and Discussion

### 3.1. Physico-Chemical Characterization

**Figure 1:**
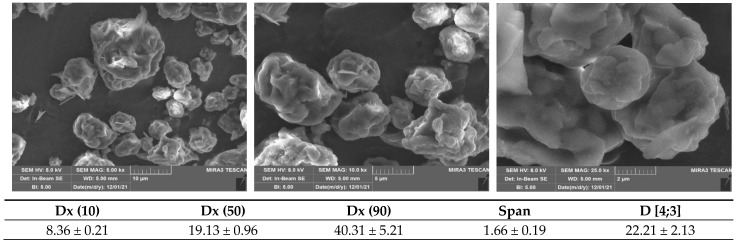
SEM micrographs (**top part**) and particle size parameters (**bottom part**) of SD crickets. The Shapiro-Wilk tests did not show a significance departure from the normality (*p* < 0.05).

Figure 1 reports the SEM images and the particle size parameters of the SD crickets. The powder is characterized by microparticles with a narrow size distribution, as evidenced by Span factor, and with 22 µm mean diameter. The microparticles are characterized by a structure with bulges and rough surfaces. In fact, these are conglomerates made of particles having dimensions lower than 1 μm.

The conglomerates should enhance cricket powder palatability, since the acceptability in food products is influenced by their perception within the mouth. By contrast, particles larger than 50–100 μm are perceived as individual entities that give a gritty feel, and their acceptability is more related to their hardness and morphology, or the characteristics of the surrounding medium and in particular, its consistency (viscosity) [13].

**Figure 2:**
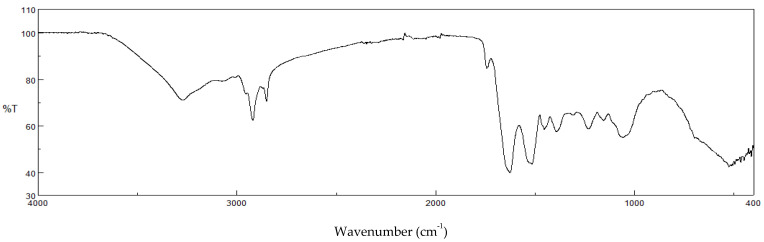
FTIR spectra of SD crickets.

Figure 2 reports the Fourier-transform infrared spectroscopy (FTIR) profiles of the SD crickets. The FTIR spectrum shows two pronounced IR absorption peaks due to its hydrocarbon chains, CH_2_ symmetric stretch at about 2850 cm^−1^, and CH_2_ antisymmetric stretch at about 2920 cm^−1^, which are ascribable to the lipids contained in the crickets.

Moreover, the bands of the amide groups should be related to the presence of chitin and proteins in crickets. In particular, an N−H stretch band around 3300 cm^−1^, amide I band (mainly due to the C=O stretch) around 1630 cm^−1^, amide II band (N−H bend) around 1530 cm^−1^, and amide III band around 1235 cm^−1^ are present. In addition to these amide bands, a broad O−H stretch band appears [18].

**Figure 3:**
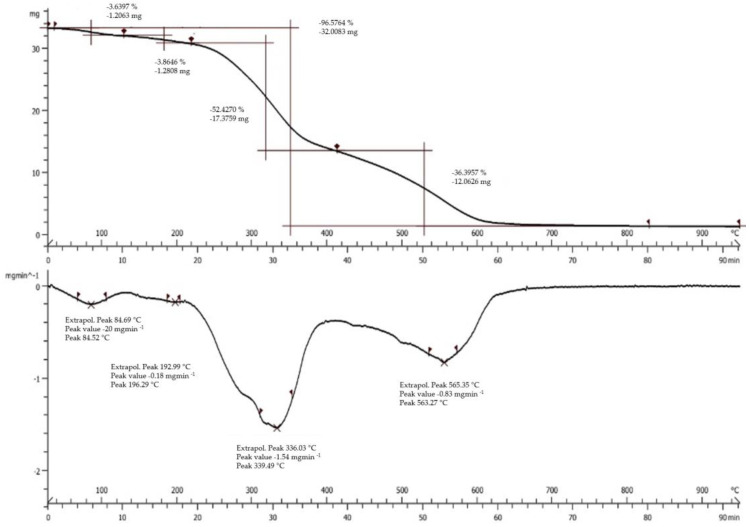
TGA (**top panel**) and DSC (**bottom panel**) profiles of SD crickets.

The thermal gravimetric analysis (TGA) curve of SD crickets is reported in Figure 3 (top panel). The TGA profile of SD crickets highlights a degradation in four stages. The first stage of the decomposition process shows the loss of free and loosely-bound water up to 180 °C (weight loss of 3.6%). The second stage of weight loss is from 180 °C to 310 °C (equal to 3.8%), followed by a third relevant stage from 310 °C to 520 °C, in which proteins and carbohydrates volatilize (weight loss of 52.4%), and finally the degradation of the resultant residues (weight loss of 36.4%).

The differential scanning calorimetry (DSC) profile of SD crickets is shown in Figure 3 (bottom panel). It could be observed that the SD crickets showed four endothermic peaks and the highest enthalpy changes occurred at 339 °C.

### 3.2. Chitin Quantification

Different methods are available to evaluate chitin content, but the acetyl group determination is more effective to quantify chitin in complex protein matrices because it directly furnishes the acetate amount on hydrolyzed samples [15]. SD crickets are characterized by an average chitin content of 3.28 ± 0.73%, equal to 32.80 ± 7.32 g/kg. These values are significantly lower than the ones reported by Kulma et al. [19] and Psaranios et al. [20], and the differences are probably related to the different life cycles of crickets used to determine chitin content. In the literature there are a few data related to chitin amount in insect species: in particular, the prepupa/pupa stages of black soldier flies, Tebo worms, Turkestan cockroaches, and house flies contain chitin ranging from 6.7 to 21 g/kg, while adult *Tenebrio molitor* and *Hermetia illucens* species contain up to 50 g/kg chitin [11], having the same order of magnitude as the SD crickets. Chitin, a polymer of N-acetyl-D-glucosamine, is the principal component of exoskeleton. It is characterized by antioxidant and antimicrobial activities and offers a wide range of applications in many sectors [21]. In humans, chitin is a fibre with defensive activity against microorganisms, and it is mostly hydrolyzed by lysozymes and hydrochloric acid found in human saliva and into the stomach [22]. Moreover, chitin is a non-digestible fiber and proven to have a positive effect towards intestinal microbiota, by acting as a prebiotic [23].

### 3.3. Surface Zeta Potential (ζ)

**Figure 4:**
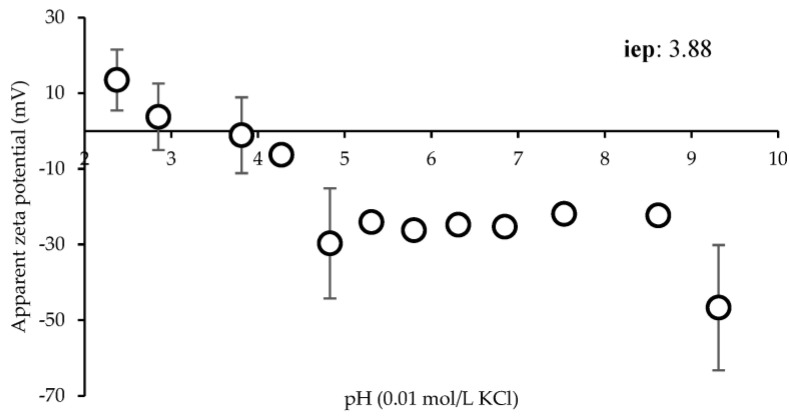
Surface zeta potential of SD crickets (mean ± s.d.; n = 3). In the inset the iep is reported.

Figure 4 shows the ζ in mV versus pH (9.0–2.0). The zeta potential changes from positive values (+13.48 ± 8.02 mV) to negative values (−46.68 ± 16.54 mV) as the pH increases from 2.3 to 9.3. From pH 2.3 to 3.8, ζ is positive, reaching the isoelectric point at pH 3.88. Moreover, ζ is negative at pH 4.3 (−6.28 ± 1.24 mV) and reaches a plateau above pH 4.8. This suggests that SD crickets should be positively charged during the gastric phase (pH 2–2.5) and negatively charged during the intestinal phase (pH 7).

### 3.4. Protein Digestability Assay

The in vitro digestibility, amino acid score, first limiting amino acid and k-PDCAAS values of cricket powder have been evaluated.

The integrity of the proteins that are being consumed and are not fecal can be assessed using the amino acid score in conjunction with protein digestibility [24]. L-tryptophan is the first limiting amino acid in cricket powder, with an in vitro digestibility of 1.08 ± 0.02 and an amino acid score of 1.04 ± 0.01. The in vitro digestibility of casein, which was used in the test as a reference protein, was found to be 0.99. Normally, k-PDCAAS values range from 1 to 0, and a score of 1 denotes a protein source with a high biological value, such as milk or eggs, which supplies the essential amino acids as indicated by the FAO. The nutritional value of the protein content in food increases with increasing k-PDCAAS. For instance, rice has a k-PDCAAS value of 0.81, vegetables have a score of 0.73, fresh and dry fruits 0.64, soy 0.91, wheat 0.42, chickpeas 0.62–0.65, and barley 0.44–0.53 [25,26]. SD crickets show a k-PDCAAS value of 1, equivalent to milk protein, eggs, and cows’ milk, indicating that this protein source can provide all the amino acids needed by humans for optimal nutrition. This could be related to the particles’ dimension and morphology. Since the SD crickets are made up of smaller particles, the proteins and consequently, amino acids’ bioavailability are enhanced and at the same level as highly nutritious food.

This method possesses some limitations. It simulates rat digestion rather than mammalian one. Moreover, it does not consider the absorption site, nor amino acids absorbed in the final portions of the intestine that are less available for protein synthesis, and it also does not take into account the concomitant presence of the microbiota that could absorb and digest the proteins. Additionally, the presence of antinutritional factors is also not considered. Furthermore, it is difficult with this test to evaluate the nutritional factor from different protein sources that could be combined in vivo, satisfying nutritional needs. However, despite this, it is recognized as a good index to obtain a prompt evaluation of protein quality.

### 3.5. Antioxidant Properties

**Figure 5:**
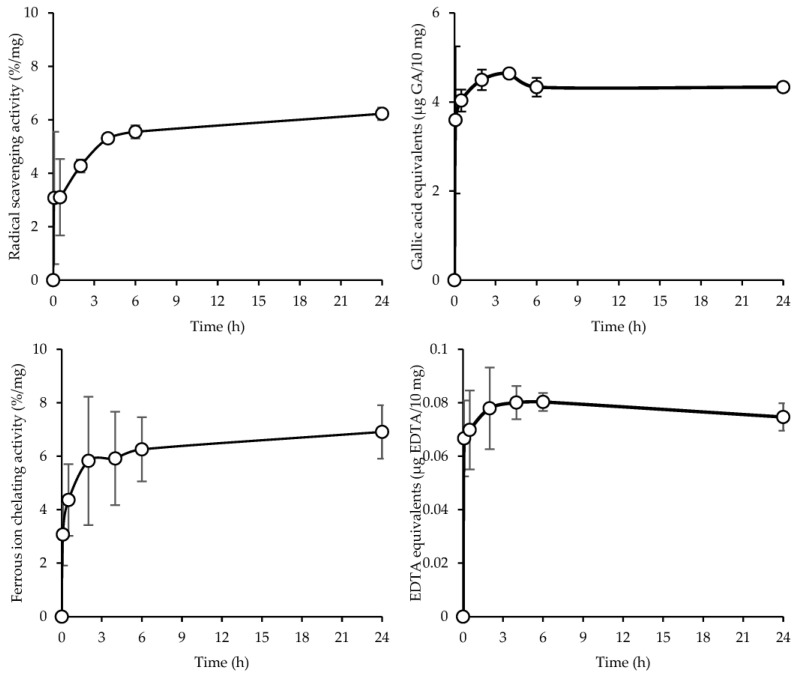
Radical scavenging activity (**top left panel**), gallic acid equivalents (**top right panel**), ferrous ion chelating activity (**bottom left panel**), and EDTA equivalents (**bottom right panel**) of SD crickets (mean ± s.d.; n = 4). Anova one-way, Scheffé test (*p* < 0.05): (radical scavenging activity) 5 min vs. 30 min: *p* = 1.0000; 5 min vs. 2 h: *p* = 0.4971; 5 min vs. 4 h: *p* = 0.0224; 5 min vs. 6 h: *p* < 0.01; 5 min vs. 24 h: *p* < 0.01; 30 min vs. 2 h: *p* = 0.5183; 30 min vs. 4 h: *p* = 0.0244; 30 min vs. 6 h: *p* < 0.01; 30 min vs. 24 h: *p* < 0.01; 2 h vs. 4 h: *p* = 0.6514; 2 h vs. 6 h: *p* = 0.4271; 2 h vs. 24 h: *p* = 0.0623; 4 h vs. 6 h: *p* = 0.09992; 4 h vs. 24 h: *p* = 0.7543; 6 h vs. 24 h: *p* = 0.9166; (ferrous ion chelating activity) 5 min vs. 30 min: *p* = 0.3159; 5 min vs. 2 h: *p* < 0.01; 5 min vs. 4 h: *p* < 0.01; 5 min vs. 6 h: *p* < 0.01; 5 min vs. 24 h: *p* < 0.01; 30 min vs. 2 h: *p* = 0.1927; 30 min vs. 4 h: *p* = 0.1444; 30 min vs. 6 h: *p* < 0.01; 30 min vs. 24 h: *p* < 0.01; 2 h vs. 4 h: *p* = 0.9999; 2 h vs. 6 h: *p* = 0.5677; 2 h vs. 24 h: *p* = 0.5131; 4 h vs. 6 h: *p* = 0.6601; 4 h vs. 24 h: *p* = 0.6060; 6 h vs. 24 h: *p* = 0.9999.

Free radicals are generated by unsuitable metabolic processes and could pose significant health problems, which can lead to a number of diseases including cancer, inflammation, and neurological conditions [11]. For this reason, the antioxidant properties of SD crickets were assessed through DPPH assay (direct evaluation), a free radical which has hydrogen acceptor capability, and via iron ions’ chelating properties (indirect evaluation), since iron is a well-known pro-oxidant which leads to the generation of free radicals from Fenton reactions. Figure 5 reports the antioxidant properties of the SD crickets as radical scavenging activity (top left panel), gallic acid equivalents (top right panel), ferrous ion chelating activity (bottom left panel), and EDTA equivalents (bottom right panel). The results show an increase in radical scavenging activity and gallic acid equivalents, and ferrous ions’ chelating activity and EDTA equivalents up to 24 h, suggesting a release of bioactive molecules which lead to an increased antioxidant capacity.

As reported by Bassett et al. [13], spray-dried crickets are an excellent source of vitamin B2, vitamin B12 and vitamin E, which act primarily as a peroxyl radical scavenger.

In addition, the antioxidant activity might also be related to the high level of proteins in crickets. It has been reported that proteins possess the capability to inhibit oxidation reactions, acting as antioxidant agents due to their capability to donate protons, chelate metal ions, and eliminate radicals [27]. These properties are principally enhanced with the presence of low molecular weight peptides having a higher level of amino acids available to interact with free radicals [28].

It is conceivable that the antioxidant activity of the SD crickets is not related to any single component, but a pool of multiple components that act with different action mechanisms and possess a synergic effect on the whole antioxidant properties.

### 3.6. Cytocompatibility and Pro-Inflammatory Immune Response

**Figure 6:**
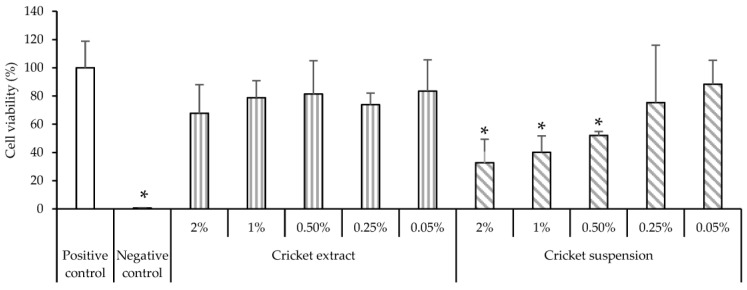
Cell viability of Caco-2 cells after 24 h of contact with cricket extract or cricket suspension (mean ± s.d.; n = 5). * indicates significant differences (*p* < 0.05).

Figure 6 reports the viability of Caco-2 cells after 24 h of contact with SD crickets. Caco-2 cell monolayer is a well-assessed model for representing in vitro enterocytes of the gastrointestinal tract. Considering ISO guidelines, the reduction of viable cells within 30% is within the biocompatibility range [29].

The SD cricket extracts show no toxic effect on cellular viability at all concentrations assayed. On the other hand, highly concentrated SD cricket suspensions show an evident decrease in cell viability. However, this could be related to the high number of insoluble particles that precipitate onto Caco-2 cells, and could inhibit the gas and nutrient exchanges of the cells in the monolayer. Such phenomena could be related not to the toxicity of the systems as better highlighted by the indirect test on extracts, but to the presence of particles in suspension that could physically impair the cells’ metabolism, leading to a decrease in cell viability. In addition, in accordance with Regulation (EU) 2015/2283, the safety of frozen and dried formulations of *Acheta domesticus* has been established [30].

**Figure 7:**
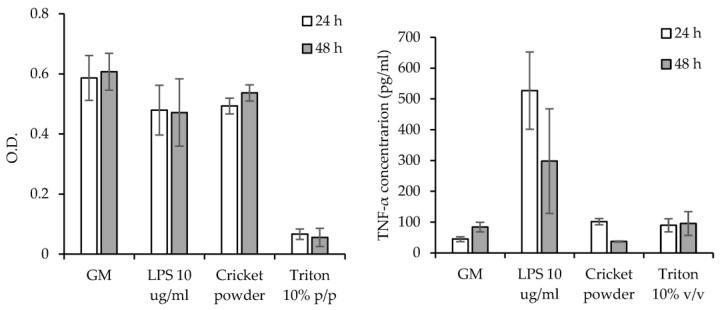
Cytocompatibility of macrophages (O.D.—optical density) after one and two days of contact with SD cricket extract (**left panel**), and TNFα cytokine expression and concentrations (pg/mL) for cells exposed to SD cricket extract (**right panel**) (mean values ± sd; n = 8). Anova one-way, Scheffé test (*p* < 0.05): (**left panel**) GM 24 h vs. LPS 24 h: *p* = 0.2441; GM 24 h vs. Cricket powder 24 h: *p* = 0.3422; GM 24 h vs. Triton 24 h: *p* < 0.01; LPS 24 h vs. Cricket powder 24 h: *p* = 0.9932; LPS 24 h vs. Triton 24 h: *p* < 0.01; Cricket powder 24 h vs. Triton 24 h: *p* < 0.01; GM 48 h vs. LPS 48 h: *p* = 0.1767; GM 48 h vs. Cricket powder 48 h: *p* = 0.6513; GM 48 h vs. Triton 48 h: *p* < 0.01; LPS 48 h vs. Cricket powder 48 h: *p* = 0.6987; LPS 48 h vs. Triton 48 h: *p* < 0.01; Cricket powder 48 h vs. Triton 48 h: *p* < 0.01; GM 24 h vs. GM 48 h: *p* = 0.7259; LPS 24 h vs. LPS 48 h: *p* = 0.9256; Cricket powder 24 h vs. Cricket powder 48 h: *p* = 0.1156; Triton 24 h vs. Triton 48 h: *p* = 1.0000 (**right panel**) GM 24 h vs. LPS 24 h: *p* < 0.01; GM 24 h vs. Cricket powder 24 h: *p* = 0.5441; GM 24 h vs. Triton 24 h: *p* = 7029; LPS 24 h vs. Cricket powder 24 h: *p* < 0.01; LPS 24 h vs. Triton 24 h: *p* < 0.01; Cricket powder 24 h vs. Triton 24 h: *p* = 0.9915; GM 48 h vs. LPS 48 h: *p* = 0.0189; GM 48 h vs. Cricket powder 48 h: *p* = 0.8348; GM 48 h vs. Triton 48 h: *p* = 0.9965; LPS 48 h vs. Cricket powder 48 h: *p* < 0.01; LPS 48 h vs. Triton 48 h: *p* = 0.0253; Cricket powder 48 h vs. Triton 48 h: *p* = 0.7269; GM 24 h vs. GM 48 h: *p* 0.01; LPS 24 h vs. LPS 48 h: *p* = 0.0567; Cricket powder 24 h vs. Cricket powder 48 h: *p* < 0.01; Triton 24 h vs. Triton 48 h: *p* = 0.7552.

Figure 7 reports the viability of monocytes-derived macrophages (left panel) after 24 h and 48 h of contact with SD cricket extracts, and TNFα concentrations (right panel) secreted by macrophages exposed to SD cricket extracts. The SD cricket supensions were not assayed since they proved to negatively influence cell growth due to the deposition of insoluble material onto cell substrates. The SD cricket extracts obtained from microparticles are cytocompatible towards monocytes-derived macrophages with viable cells comparable to the positive control (growth medium—GM), and significantly higher than that of Triton (10% *v/v*), used as negative control. As for TNFα concentrations (right panel), the SD cricket extracts do not show proinflammatory activity, with a TNFα secretion significantly lower than that stimulated by LPS (positive control) and superimposable to the negative control (GM).

## 4. Conclusions

Crickets have generated great interest as a novel source of bioactive molecules for human nutrition. In this work, the properties of a novel Italian SD cricket powder were successfully characterized, highlighting its potential as an innovative tool to be used in both food and medical fields.

The SEM characterization demonstrates that the powder is composed of particles with microdimensions and rough surfaces, made up of conglomerates of smaller dimensions. Moreover, the dimensional analysis evidences a narrow size distribution with a mean diameter of 22 µm that should enhance palatability, and consequently the compliance of the final consumer. The solid state characterization highlights the presence of lipids, proteins and chitin. The amount of chitin was also evaluated, since it is a bioactive polysaccharide characterized by remarkable antioxidant and antimicrobial activities. SD cricket powder has an average chitin content of 32.80 ± 7.32 g/kg. This could contribute to a wide range of applications across a number of fields, including protection against inflammatory and neurodegenerative diseases.

The zeta potential evaluation demonstrates that the surface charge changes in relation to the environmental pH after ingestion. Notably, the values changed from positive to negative as the pH increased from 2.3 to 9.3, reaching the isoelectric point at pH 3.88. This suggests that SD crickets should be positively charged during the gastric phase (pH 2–2.5) and negatively charged during the intestinal phase (pH 7).

The in vitro protein digestibility shows that the SD cricket powder is characterized by a k-PDCAAS value of 1, indicating it is a protein source that could provide all the amino acids needed by humans for optimal nutrition. This could be related to the fine particle size that favours bioavailability. Moreover, the antioxidant assays show an increase in both radical scavenging activity and ferrous ions chelating activity up to 24 h, suggesting that the powder possesses antioxidant activity. This particular aspect could prove of great interest to the medical field, since free radicals generated by altered metabolic processes could cause significant injuries, and lead to a number of diseases including cancer, inflammation, and neurological conditions.

The SD crickets show no toxic effect on cellular viability towards both Caco-2 cells and monocytes-derived macrophages. Moreover, the SD cricket extracts do not show pro-inflammatory activity.

In conclusion, the investigations into SD crickets underline their potential nutritional value since they may be considered equivalent to milk protein, eggs, and cows’ milk, all highly nourishing foods. Moreover, the antioxidant properties, biocompatibility, thermal stability, and anti-inflammatory properties support the potential use of SD crickets in the food industry by providing health benefits in addition to their basic nutritional value. These results are confirmed using different batches, showing a low variability of the SD crickets’ properties from one batch to another. The consistency of the batches and their properties prove that SD crickets represent an interesting material and future application could be envisaged, such as their use in tissue engineering for their outstanding antioxidant and anti-inflammatory activity.

## Data Availability

All of the data is contained within the article.
